# Clinical Correlates of Hwa-Byung and a Proposal for a New Anger Disorder

**DOI:** 10.4306/pi.2008.5.3.125

**Published:** 2008-09-30

**Authors:** Sung Kil Min

**Affiliations:** Department of Psychiatry, Yonsei University College of Medicine, Seoul, Korea.

**Keywords:** Hwa-byung, Anger, Anger syndrome, Anger disorder

## Abstract

This paper reviewed the studies on hwa-byung (HB), which literally means anger disorder and this is known as the culture-related chronic anger syndrome of Koreans. Based on these studies and a review of the literature on the anger syndromes of other cultures, I have proposed a new anger disorder. The rationale for this proposition is first that the clinical correlates of HB, including the epidemiological data, the etiological factors, the symptoms and the clinical course, are unique and different from those of the depressive disorders, which have been postulated to be similar to HB. Second, the symptoms of HB are characterized by pent-up anger and somatic and behavioral symptoms related to the release and suppression of anger. Third, a group of patients with only HB and who visit psychiatrists for treatment have been identified. Fourth, anger is thought to be the basic target of treatment for HB patients. Last, anger syndromes like HB have been identified, with various names, in other cultures. By reducing the cultural variation of HB and integrating the common clinical correlates of the syndromes related to anger, a new anger disorder for the mood of anger can be conceptualized, like that for other mood disorders for the corresponding pathological moods. The research diagnostic criteria for HB and the new anger disorder are also suggested. I propose that the new anger disorder to be included in the new international classification system as a member of the larger family of mood disorders. International collaborative studies are needed not only to identify such anger disorder in various cultures, but also to explore giving better treatment to these patients based on the bio-psycho-social model of anger disorder.

## Introduction

Hwa-byung (HB), which literally means "anger disorder" or "fire disease", is known as a Korean culture-related syndrome. Hwa means anger and fire as well, and byung means disease or disorder. HB is also explained as being abbreviated from "wool-hwa", meaning dense anger-fire or pent-up anger. The term HB has been used by lay Koreans and it is not found in the classical textbooks of traditional oriental medicine.

HB has been discussed since the 1960s among Korean psychiatrists in regard to its concept, its culture-relatedness and the Diagnostic and Statistical Manual of Mental Disorders (DSM) diagnosis. Systemic studies by psychiatrists on this syndrome have been conducted since 1977 and these have been published only in the Korean medical journals.^1^-^20^ These studies have been done on the concept, epidemiology, symptoms, etiology, dynamic considerations, DSM-III or DSM-IV diagnosis, treatment and prognosis of HB to gather information so as to help inform Korean psychiatrists. Meanwhile, HB has been studied by researchers of oriental medicine since 1992,^21^-^24^ with the collaboration of clinical psychologists.^25^-^28^ Their reports reconfirmed the results o f psychiatrists' previous studies regarding the disease incidence in terms of gender and age, and the psychogenecity as related to anger and symptoms, though their basic ideas on the disease-model and treatment methods were different according to the different theories of oriental medicine. HB has been also widely studied by nursing scientists,^29^-^36^ and these studies focused on women's health in the cultural context of Korean society and nursing care for treating the anger of patients with chronic diseases and also their care-givers. Counselors have also studied HB to understand their clients who have HB or anger problems.^37^-^40^ Most of these studies have been conducted on patients with self-labeled HB. HB was firstly introduced to English speaking countries as a case report by Lin in 1983,^41^ and this was followed by clinical studies;^42^-^44^ HB was then listed in Appendix 1, the Glossary of Culture-bound Syndromes in the DSM-IV in 1994.^45^ Roberts et al.^46^ reported the development of an MMPI-2 scale designed to assess the features of HB. Biological studies of HB are now ongoing.^23^,^47^

The diagnostic criteria of HB^24^ and the Hwa-byung Scale^28^ have recently been proposed by a group of oriental medical physicians, clinical psychologists and psychiatrists. Studies^18^,^19^ using this diagnostic criteria have revealed almost the same results for the DSM-IV axis I diagnosis of HB as the DSM-III axis I diagnosis of self-labeled HB in the previous studies.^2^,^3^,^9^,^13^ This means that the results of studies on the other clinical correlates of self-labeled HB might well represent the HB that is diagnosed according to clinically defined diagnostic criteria. However, the clinical correlates of HB need to be reconfirmed by research that uses more appropriate diagnostic criteria for HB, and I suggest these diagnostic criteria in this paper.

The studies on the clinical correlates of HB have been reviewed once.^17^ In this paper, I examine other studies on the clinical correlates of HB, and based on these studies and the related literature on anger syndrome in other cultures, I have conceptualized a new anger disorder and we propose that it be included in the new international classification system of mental disorders. I also suggest new research diagnostic criteria for HB and anger disorder as well.

## Clinical Correlates of Hwa-Byung

### Concept of hwa-byung

HB is supposed to be a disorder that is basically related to anger, though the hwa in HB has been variously described by ordinary Koreans as anger/fire, stress, emotion, conflicts or fire or something accumulated in the body.^5^ The anger in HB is thought to be a reaction to unfair social violence.^1^,^4^-^8^ According to the patients' explanation, they have to suppress anger so as not to jeopardize harmonious social relationships. But if an angerprovoking situation repeats itself, then the suppressed anger accumulates and becomes dense, which is usually describes as wool-hwa, which means dense hwa (anger of fire), and then HB finally develops.^5^

Anger has been also identified as a basic symptom of HB. For HB patients, the symptoms represent partially suppressed and partially expressed anger. The characteristic symptoms of HB seem to be unique and different from those of other mood or affective disorders like anxiety or depressive disorders because the characteristic symptoms of HB are basically related to anger.^1^,^4^-^8^ Chon et al.^25^ suggested that anger (trait anger and state anger) was more highly rated by patients with HB than that by the healthy controls, and HB was more significantly related with anger than with depression.

Here is a typical case.

#### A Case History

The patient was 49 year old housewife. She came to us with the chief complaint of pent-up anger, "hwa", which was intermittently accompanied by a hot sensation, that had to be cooled with a fan, and something pushing-up in her chest. The other symptoms were "many things accumulated" in the epigastirum and respiratory stuffiness that used to be relieved by frequent sighing. Some times she used to feel so angry and so "ukwool" (a feeling of unfairness) that she felt almost like loosing control or becoming crazy. Her self diagnosis was hwa-byung. The reason for her anger was her family situation with her husband and mother-in-law. Her anger began 15 years ago just after her marriage, when she realized that she had been deceived by her husband regarding his past history, including his lack of education and poorer economic condition than what he claimed before marriage. Since then, she lived in an angry mood with the frustration related to a hard life. Moreover, her mother-in-law had to live together with them, as her husband was the first son, and she began to treat her unfairly and everyday the mother-in law unfairly stepped into her private and marital life. She had to suppress her anger and hide the hatred and obey her husband and his mother to keep peace in the family. While living with her husband, she found her husband to be a truly good man, and that is why she kept their marriage intact until now. Nevertheless she gradually became irritable and nervous. She recently became more irritable, began to beat her husband, throw things at the children or abuse them. She said the children never understand why she was so angry. When she recently stood up against mother-in-law for the first time, she felt "cool" for a while. During the interview, she talked extensively with sighing and tears about how she had suffered from a life of "ukwool and boon" and with "many haan" ("ukwool" and "haan" will be described later). But she said she did not feel depressed and had never thought about suicide. Rather, she has tried to live enthusiastically and actively, and she regularly attended her work place (cleaning buildings). She has tried to avoid being isolated from her fellow workers and she believed they might think of her as a "good" person. She revealed her painful past memory that she had been discriminated against by her mother for being a daughter and her mother favored the sons, and she had been frequently beaten by her mother and older brother during her childhood. Sometime she had to escape under a table to avoid being beaten. Her mother did not even bring her to the hospital when she had broken her leg. She said her childhood was full of days of tears and her life was "full with many haan". Naturally, her explanation for her illness was "accumulation of anger (hwa)". Her history revealed most of the characteristic components of HB, including chronic suppression of anger, "haan", women's issues, typical symptoms and the Korean traditional culture in her family life.

As in this case, three emotional expressions of anger seem to be key concepts to describe HB, and these are etiological factors and symptoms as well: "hwa", "ukwool and boon" or "haan".^4^,^5^,^10^,^14^ It is difficult to translate these Korean words into English.

##### Hwa

Hwa simply means "anger" and fire as well, but some patients describe it as heat or "a mass of heat" in the body, or something that has "accumulated" in the chest/abdomen. Koreans used to think that if anger, negative emotion and/or the stress in social life are repeated, then they accumulate and become dense, "woolhwa", and this is transformed into a mass in the epigastrium.^4^,^5^ Koreans seem to be sensitive to anger in the traditional, social and cultural context. There are many words accompanied by hwa as a prefix or a suffix in Korean. For example, hwa-sul means much alcohol drinking to calm down anger. Hwa-gim means "in an angry mood" or "because of anger" (people sometimes set fire impulsively in hwa-gim, or they impulsively agree to a divorce in hwa-gim). Shim-hwa means fire in the heart, and this is anger. Koreans use many words in everyday talking and these words contain the physical nature of fire to describe various state of anger. Examples are heat, hot, boiling, burning, flaming or rising-up (pushing.).

In Korean oriental medicine, hwa has many meanings, including not only anger and hwa (fire), but also functional excitement (arousal) of hwa-ki, one of universal 5 "ki"s (qi in China). Hwa-ki (ki of fire) is supposed to be internal heat caused by one of various emotions attributed to different organs, including the liver, heart and kidney and when this is excessive, it may damage organs and lead to diseases.^21^

##### Uk-wool and boon

These two words are frequently used together and are they used to refer to an individual's perception when their desires are blocked by unfair and wrong social powers. "Uk-wool" is a feeling of anger as a victim, and in a Korean-English dictionary,^48^ this is translated as "vexed", "mortified", "regrettable", "victimized", "suffer unfairness", "falsely accused" or "mistreated". "Boon" is a feeling of anger arising from failure due to indefensible external reasons, misfortune or a slight lack of ability to achieve final success. "Boon" is translated as "resent", "exasperate", "indignant", "mortified", "vexed", "chagrin" or "sorry". In this paper, this term will be expressed as "a feeling of unfairness".

##### Haan

The mood described by 'haan' is complex and may have some negative components, a mixed feeling of missing someone, sorrow, regret, sadness and depression, along with some feelings of hatred and revenge. In the Korean-English dictionary, "haan" is translated into English as "grudge", "rancor", "spite", "regret", "lamentation", "grief " or "hate". Haan may mean "everlasting woe".^49^ When haan has a component of longing for a lost loved one, it is called "jeong-haan". When it has a component of hatred and revenge, it is called "won-haan", When it has a component of regret, it is called "hoe-haan". Tong-haan means painful haan. Kim^50^ related won-haan to HB.

Haan has been considered as being the result of personal or collective trauma, unsatisfied desire, suppressed anger or uk-wool/boon. The mental mechanisms and coping strategies related to haan include somatization, suppression, splitting-projection, passive-aggressiveness, oral consumption, primitive idealization, stimulus reduction, self-pity, shared-concerns and dependency. Some of them (somatization, suppression and oral consumption) are common in people with HB.^11^,^51^

Haan is known have a positive side as well, in which haan may be transformed or sublimated into positive energy. The emotional power of haan may encourage one to keep living or surviving (usually described as "living with harboring haan") or to engage in creative activities.^52^-^56^ For example, the haan of a mother who has suffered from poverty, less education, a violent husband or a harsh mother-in-law can finally be solved (haan-puri) a long time later by the success of her son or daughter. It is comparable to making kimchi, a traditional Korean vegetable, which is made by fermenting vegetables for a long time with hot red chili in a closed pot.

##### Collective haan and Korean culture

Many scholars consider haan as a unique Korean sentiment beyond its literal meaning, and it is a key word to understand Koreans or Korean culture. Haan has been thought of as Koreans' traditional, cultural and collective emotional state of suppressed and accumulated anger or uk-wool. Koreans have endured repeated suffering from both domestic and international injustice and unfair violence throughout their nation's history.^53^ Ordinary people, farmers, servants or other people of the lower class were suppressed by bureaucrats or the literate upper class, called yang-ban. Women were suppressed by men. But haan has been the source of energy for the creativity of ordinary people including for example, the ceramic art that has been made by unknown masters, or for making revolution against political suppression including, for example, a farmers' military rebellion against the local government in the 19th century, which is called Donghak-ran. The typical collective Korean experiences with haan in modern history include the inherited poverty for thousands of years, Japanese colonization, the Korean War and division of the country, suppression by military dictators and the recent economic polarization. But haan has been also thought to provide energy to Koreans for economic development (haan-puri of poverty) and democratization (haan-puri of political suppression) during the so-called "condensed history" of Korea. The haan of women has been solved by women's liberation.^52^,^56^ Accordingly, Korean history is referred to as a history of haan and Korean culture as a culture of haan (In this paper, haan will not be translated and it will be used as it is.).

##### Jeong

HB has been explained in connection with jeong, haan and the we-collectivism of Koreans.^15^,^20^ Jeong is another Korean term for culture-related affect, and it may be defined as a complex of loving, liking, caring, bonding and attaching that is developed through long person-to-person or person-to-object relationships, like cathexis, and its nature is described as adhesive or sticky. For example, in Korea, mother-love used to be called mother-jeong, friendship as woo-jeong, love for a lover as ae(love)-jeong or yon(longing)-jeong, and love for the hometown as jeong for the hometown. When jeong is cut or detached by a lover, the resulting feeling is usually called as jeong-haan. Chung^20^ thought that haan developed when jeong was violated, and he discussed the relationship between jeong, haan, HB and the "we" collectivism of Koreans. Another author speculated on the relationship between jeong, haan, HB and the ancient philosophy of "han" (meaning one, big and brightness).^15^

### Epidemiology

In an epidemiological study of a rural area of Korea, 4.1% of the general population was reported to have HB.^9^ HB is reported to be frequently found in middle-aged or older housewives of the lower social class.^4^,^9^,^18^,^19^ Chon et al.^25^-^27^ suggested that HB patients know the importance of anger as the etiology of their illness. A high prevalence of HB in middle-aged or older housewives in the lower social class was confirmed by studies on patients with HB who were diagnosed by the diagnostic criteria of HB.^18^,^19^ Generally, HB is an illness of Korean women who suppress their anger that's the result of family conflict so as not to jeopardize harmonious family or social relationships, which are highly valued in traditional Korean culture.^33^-^37^

### Etiology

#### Psychogenecity

The etiology of HB has been described by patients as being anger and/or anger-related negative emotional reactions, including "uk-wool", "boon" or "haan", and these have been accumulated for a long time.^4^,^5^ A study on Korean women's perceptions of the life situations that cause HB revealed three situations: a vulnerable situation (for example, chronic poverty), lowered self esteem (for example, unfair treatment by the father and/or husband), and negative life events (for example, trauma and an unfortunate fate).^36^ Those Koreans who have ever experienced HB usually admit that their HB is a disorder of a psychological origin, which means it is a psychogenic or reactive disorder.^5^,^12^ These results on the etiology of self-labeled HB were almost same as those results for the patients with HB and their HB was diagnosed with using the diagnostic criteria of HB.^18^

#### Precipitating Factors

The most common precipitating source of this syndrome are unfair social situations, which were typically and most commonly related to the violence of husbands and mother-in-laws toward housewives.^1^,^4^,^5^,^18^,^33^-^38^ Many Koreans with HB feel that they are the victims of chronic unfair social aggression, which is related to the traditional culture of the patriarchal Korean social system and the fatalism and collectivism of Koreans.^35^ Other precipitating factors include poverty, which used to be considered as a result of unfair social deprivation, various forms of unfair social aggression or suppression or social injustice, which include violence, discrimination, isolation, exploitation, failure in business or failure to be promoted, loss of property, unfair juridical decisions, betrayal or swindling.^4^ Probably the most tragic unfair trauma that was caused by social aggressors and that some of Korean women had ever experienced might be the trauma of sexual slavery for Japanese soldiers during WWII (comfort women).^57^ The former comfort women are still so angry, ukwool/boon and "many haan" and most of them call their illness HB.

#### Predisposing Factors

HB is considered to be an illness of women.^1^,^4^,^5^,^33^-^38^ Regarding age, HB is more frequently found in middle or older age people.^4^ Genetic studies on HB have not yet been conducted. Yet a study^9^ on family history revealed that first degree family members of patients with HB had more frequent "general neurotic" disorders ("nervous" or "noirose" in Korean, including depression and anxiety disorders), insomnia, personality problems, violence and suicide as well as HB than those first degree family members of persons without HB. When patients with HB were evaluated with using the Korean version of State Trait Anger Expression Inventory (STAXI),^26^ the results showed that trait anger as well as state anger was highly related to stress in the patients with HB, and that in the early period of HB development, anger-out was predominant and in the later period, anger-in was predominant and that trait anger and angerin were significant predictive factors for HB. Negative childhood experiences or trauma have been explored for their relationship with HB,^4^,^5^ and these included parental abuse and neglect, isolation among peers, general discrimination, sex discrimination, and bullying (ijime) by peers. These experiences might have installed inferiority, damaged self-esteem or even feelings of being persecuted or being a victim and a chronic paranoid tendency in children's minds, and these feeling may last until adulthood and make these individuals sensitive, vulnerable and likely to become angry and easily provoked by trivial things.

#### Pemorbid Personality

If HB is understood as being the product of the interplay between external unfairness and internal responding, then research should be done on the personality characteristics of patients with HB. Min et al.^4^ suggested that their characteristics were being hasty (typically described as "fire-like" or "convulsive"), timid, perfectionistic, sensitive and introverted, and they have poor social skills. Low self-esteem has been suggested as an important causal factor of HB, and especially for middle-aged women.^31^,^36^ Kwon et al.^28^ reported that the personality characteristics related to HB were not different from those related to depression, although the symptoms were different. Roberts et al.^46^ reported from their research using the MMPI-2 that HB was most highly correlated with Hy-O (hysteria-obvious, which measures the development of physical symptoms in response to stress), Hs (which measures somatic complaints), and HEA (health concerns). Principal component analysis of the scale items revealed four components: general health, gastrointestinal symptoms, hopelessness and anger. Hwang^58^ related HB to the narcissistic/masochistic personality of the patients.

#### Psychodynamic Explanation

HB seems to begin with anger and it develops into a syndrome that's complicated with its suppression, accumulation, partial behavioral expression and somatization.^4^-^6^,^10^ The styles of defense and the coping strategies relating to HB included suppression-inhibition-withdrawal, somatization, oral consumption, avoidance of stimulus, externalization (projection), help-seeking complaining, impulsiveness (acting-out), pseudoaltruism, omnipotence, self-pity, fatalism and fantasy.^11^ Similarly, another study^26^ suggested the coping styles HB patients employ include active forgetting, accommodation, fatalism, emotional pacification, emotional social support seeking, passive withdrawal, self-criticism, perseverance, seeking problem-solving social support and seeking religion, and that HB patients had a higher tendency to suppress their anger (anger-in).

These findings can help determine what kind of persons (oral consumption, pseudoaltruism, omnipotence, self-pity, fatalism, fantasy, self-criticism and perseverance) develop HB, why they develop HB (avoidance of stimulus, help-seeking, emotional pacification) and how they develop HB (suppression-inhibition-withdrawal, accommodation, somatization, projection, active forgetting, impulsive acting-out). Suppressed anger may cause somatic symptoms that symbolize the suppression of anger-fire. Anger expression may appear directly through feelings of a angry mood, temper, physiological reactions of anger, behavioral anger expression and/or somatic symptoms that symbolize releasing of anger. Differences were found in the expressed symptoms between HB and depressive individuals in spite of their similar personality characteristics, suggesting there are differences in the selective adoption of different defense mechanisms.^28^

HB appears to be like an inactive volcano, from which fire, smoke and lava are partly leaking. In this metaphor, an anger attack or intermittent explosive disorder are like an active volcano with an explosive eruption of flame and lava, and haan is like an extinct volcano with lake that may look peaceful and beautiful though under which flame is ready to erupt.^5^

##### Theoretical consideration

The dynamic relationship between anger, depression and anxiety has been discussed since Freud.^59^,^60^ Basic human emotional reactions, including sadness, anger, anxiety/fear and pleasure, are known to be related to basic instincts (libido and aggression). Among the basic negative human emotional reactions, anger seems to be the most primary one. When instinctual desires are frustrated, anger may primarily develop. This primary anger may be observed more primarily than anxiety or depression in animals and young children when they are frustrated with basic instincts. As a human grows, emotional reactions become more differentiated into depression and anxiety and then to hate, guilt, shame or disgust. In mature human adults, anger usually has to be suppressed, defended or coped with when confronting social needs and it may be expressed in various disguised forms so as not to jeopardize social or interpersonal relationship.

Accordingly, in patients with HB, their subjective anger might have been complicated during the chronic course of suppression into a syndrome formed according to the individual's predisposition and selective adoption of defense mechanisms and coping strategies. Anger may be defended in the various forms, with most common probably being depressive disorders. Depression has been explained as being the result of repression of anger or turning anger/aggression turning toward him or herself. Anxiety seems to be a feeling for impending disaster due to an explosion of anger. Anger may be defended with somatization that results in pain, mass formation and respiratory stuffiness, as in HB. When anger and aggression are expressed directly in an excited form, it may easily be accompanied with an elated mood such as hypomanic or a manic state. Further, anger, aggression or the feeling of being a victim may be suppressed and displaced or projected to outside, resulting in suspicion and/or a paranoid state or it may provide energy for revenge. Anger may be sublimated to positive energy for the will to live, productivity, artistic creativity or social reformation.

##### Korean oriental medicine

Researchers of Korean oriental medicine had speculated on HB theories related to the concepts of "organ ki" and yin-yang: stagnation of vital energy ("ki") in women, depression of liver-energy (ki), imbalance between heart-yang and kidney-yin, fire-ki caused by long-term suppression, fire syndrome caused by an excess of five emotions or the deficiency of yin leading to fire.^21^ These explanation relating to the concept of femininity, stagnation or depression of vital energy, fire and heart-mind seem to be partly in line with the psychodynamic concept of HB.

##### Sick role

As HB has been formed in the social context, almost all the Koreans around HB patients know what kind of illness these patients are suffering from and why they become ill. When a housewife becomes known to have HB, which may be self-labeled, or labeled by a neighbor, a physician, people around her or a shaman recognize that she is suffering from an unfair familial or social situation and they try to help her not only by recommending various treatment, but also by advising what she, her husband, her mother-in-law or her other relatives should do, for example, providing "care" in time.^37^ Usually the husband or mother-in-law begins to change their attitude toward the patient, if they are not too rigid a person. The previously mentioned defense mechanisms and coping strategies such as help seeking^11^,^26^ and the personality of hysteric-obvious^46^ seem to be related to the sick role of HB patients.

#### Biological Research

There is little research on the biology of HB. But a study using digital infrared thermographic imaging reported that the temperature on the body of HB patients was highest in the anterior middle chest and the difference in temperature was biggest between the upper and lower back.^23^ Lee et al.^47^ recently reported that in a study on neural responses to neutral, sad and angry facial stimuli with using functional magnetic resonance imaging (fMRI), HB patients showed increased activation in the lingual gyrus and fusiform gyrus as compared with healthy persons, but there was relatively lower activation in the thalamus, and lower activity in the responses to the neutral condition in the right anterior cingulate cortex than that in the healthy controls. They suggested that suppression of affect results in aberrant function of the brain regions of the visual pathways, and functional impairment in the anterior cingulate cortex may contribute to the pathophysiology of HB.

### Symptoms

HB is characterized by anger and anger-related emotional, physiological and behavioral symptoms.^1^,^4^,^5^,^14^ The results of the studies on the symptoms of patients with self-labeled HB are almost same with those for the HB patients with HB diagnosed by using the diagnostic criteria of HB.^18^,^19^ The somatic and behavioral symptoms seem to have culturally-related symbolic meaning: they symbolize the nature of fire and heat or fire-ki in the body organs and/or the suppression of these elements.

The basic symptoms seem to be psychological symptoms, including subjective pent-up anger (hwa) and a feeling of uk-wool/boon (a feeling of unfairness, and the somatic symptoms of a heat sensation.

Anger used to be frequently felt as "appearing or developing from inside", and this pent-up anger is experienced either persistently or intermittently with a hostile feeling toward the persecutors. Intermittently experiencing HB symptoms such as palpitation, heat sensation and respiratory stuffiness seems to be like attacks, which are sometimes so serious that the sufferer almost looses control, like a panic attack. But these HB attacks may develop spontaneously or on trivial provocation or upon remembering past events or persecutors. HB attacks, in a difference from panic attacks or anger attacks, usually have a rather slow onset and they disappear more slowly. Other related psychological symptoms are many thoughts, and feelings of "haan", anxiety with agitation, a sad mood that is frequently accompanied by tears and sighing, a pessimistic or nihilistic mood, and a feeling of guilt.

Another typical symptoms of HB are physiological signs of anger and excitement. Most typical is a heat/hot sensation in the body, which appears mainly in the upper trunk, and this may also be accompanied with hot flushing, a feeling of boiling inside, sweating, intolerance to a hot environment (patients usually go-out to feel cool outside). Other typical somatic symptoms include palpitation and dry mouth. Other less frequent symptoms include insomnia, anorexia, tremor and cold sweating. Sometimes a cold sensation and cold sweating on limbs is accompanied with a heat sensation in the trunk.

The somatic symptoms seem to symbolize the expression or suppression of anger-fire. Respiratory stuffiness (chest oppression or chest tightness) seems to symbolize suppression of anger (symbolizing the nature of pent-up smoke from a suppressed fire). Sighing seems to symbolize relief from suppressed respiration. Something pushing-up in the chest seems to symbolize the nature of anger-fire developing acutely from deep inside of the body-mind. A mass in the epigastrium or chest seems to symbolize the suppression, accumulation and increased density of anger-fire. Headache and pain seem to symbolize psychological distress.

The behavioral symptoms includes expressing anger such as irritability, a temper tantrum, abuse, throwing things or quarreling, impulsively going-out from a closed or hot room or situations (usually to feel the cool air), tears due to being so angry and much pleading (talkativeness or "hasoyeon" in cultural Korean terms), and this is usually accompanied by frequent crying, sighing, lamenting and even paradoxically vacant smiling. This kind of talking is also known as typical behavior related to "haan", and it is very hard to stop during an interview.

The general neurotic symptoms, including a depressive mood and anxiety with agitation, frequently accompany HB. A depressed mood in an HB patient is characterized by a sad or nihilistic mood with tears of anger (the patients used to say they weep because of anger and uk-wool rather than because of a sad mood). Patients with HB usually deny suicidal ideas, but rather, they express a strong will to live. Typical HB patients work hard, eat well, sleep well and associate with others well (as seen in case history). The anxiety is also characterized by agitation but rarely by a feeling of loosing control, from which western psychiatrists might get the impression of panic attacks.^41^,^45^

Kwon et al.^18^,^28^ reported that patients with HB diagnosed by the diagnostic criteria of HB^23^ complained more frequently of symptoms than did the patients with depression and that these symptoms included respiratory stuffiness, an epigastric mass, a heat sensation, pushing-up, ukwool/boon, subjective anger, palpitation, headache, being easily frightening and haan, and that the characteristic symptoms of HB patients were different from those of patients with depressive disorders. In HB patients, the scores for somatization, obsessive-compulsiveness, interpersonal sensitivity, fear/anxiety, hostility and paranoid symptoms on the Symptom Check List-90 (SCL-90) were significantly higher than those for the patients with depression, but the scores for depression and psychoticism were not higher for HB patients. These two researches suggest that HB is a more severe illness than depression, as the HB symptoms usually have superimposed depression. This finding is compatible with the report of Lin et al.,^41^ in which the symptoms of HB patients were more severe than those of depressed patients.

### The clinical course and prognosis

HB is known to be a chronic syndrome with the mean duration being longer than 10 years (range: 7 months to 46 years).^4^,^16^

Lee,^1^ in his papers, proposed that HB developed through 4 stages, namely ① shock, in which hwa-ki or hot temper is predominant and this manifests with anxiety and irritability, ② conflict, ③ resignation (giving-up), in which a bad temper is predominant and ④ finally haan remains and persists. Chon et al.^25^ suggested that the expression of anger was predominant in the early stage, while anger suppression was predominant in the late period. However, Min^5^ suggested that HB is a mixed form of these various conditions of angry feelings, "uk-wool/boon", conflict, irritability, resignation and haan. This means that for HB, a part of the anger is suppressed, while the other part is partially or indirectly expressed, and such a state chronically persists. This idea is supported by another scholar.^61^ In the long term course, depression, anxiety and somatization disorders, which might have developed depending on individuals' predisposition and defense mechanisms, may be comorbid with or superimposed on HB.

### Treatment

Various treatment modalities have been suggested for this culture-related anger syndrome.^9^ The target of treatment has been, of course, reducing the anger.

HB has traditionally been managed with psycho-social treatment, including so-called "puri" (solving), "hwa-puri" (solving anger, usually expressed as temper or aggressive acting-out), or "haan-puri" (solving haan, usually meaning wish-fulfillment). Technically, they include talking-out or much pleading (hasoyeon), dialogue, debriefing of traumatic experiences, catharsis or abreaction or sometime obtaining revenge. Therefore, Koreans have developed many cultural or folk methods for helping patients with HB. A mask-dance, madang-theater, pansori, folk songs,^29^ or meditation^40^ have been practiced to solve people's anger, HB or haan. Traditional herb physicians have prescribed herb medicine or acupuncture that's given at certain designated points.^22^ Shaman rituals called as "sal-puri" (solving evil or damnation) have been used and this is still popular for treating HB, in which HB is supposed to be caused by the harm inflicted by a evil spirit of a soul who died with uk-wool and haan, and especially won-haan.

#### Help-Seeking Behavior

To relieve pent-up anger, ordinary people prefer and recommend talking to others. Women with HB typically are supposed to talk to their mother or sister or their friends about their anger, and especially the anger relating to the husband and his family. For educated or young people, professional treatment usually means psychological approaches (so-called counseling), while older people prefer pharmacological or medical treatment.^6^ Before coming to see a psychiatrist, many HB patients had already attempted an average of 2.4 treatment modalities.^4^ Most of them had visited physicians other than psychiatrists (70%), traditional oriental physicians (55%), and other psychiatrists (20%).

It is noteworthy that some patients sought help from Christian faith healing (confirming prayer)(12.5%) and shaman rituals ("goot")(7.1%).

#### Psychotherapy

Korean psychiatrists recommended combined psychotherapy and drug therapy for "anger reduction", and general medical treatment for the somatic symptoms.^6^ Lee^1^ recommended short-term supportive psychotherapy and catharsis, but not necessarily psychoanalysis. However, psychoanalysis need not to be excluded.

Anger management or cognitive-behavioral therapy may be used for the control of anger. Interventions for the purpose of anger reduction should target different aspects of the cognitive-emotional-physiological experiences, the relationship of anger to its triggers and/or the behavior or thoughts.

HB is so closely related to an oppressive family that family intervention is needed as well. Family therapy (or couple therapy) may be the most important treatment modality^39^ because the HB of women is considered as being caused by conflictual relationships with their husbands and/or their mothers and sisters.

Various art therapies, including music, painting or dancing, have been attempted to reduce the anger and symptoms of anger syndrome. Folk art may be better applied for the treatment of HB^29^ as HB is related with traditional culture. Religious methods, including forgiveness, meditation and praying, have been recommended by pastoral counselors.^58^,^62^

#### Pharmacotherapy

Much information on the biological or neurochemical nature of anger has been accumulated, but the information for the treatment of anger problems is not yet sufficient. There have not yet been any systemic clinical trials for the drug treatment of HB. However, most Korean psychiatrists (78.5%) recommended the combined use of antidepressants and antianxiety drugs for HB.^6^ These treatment methods remain to be proved by systemic scientific research. Based on a review of the drug treatment for the aggressive behaviors, impulsivity and violence of various psychotic disorders, including intermittent explosive disorder, aggressive behaviors in children and adolescents and especially anger attacks in major depressive disorder, antipsychotic drugs, anti-anxiety drugs, antidepressants, anticonvulsants, lithium, beta-blockers and SSRIs may be recommended for controlling anger and its physiological arousal,^63^-^68^ although aggression, impulsivity or violence may not be just the same as subjective anger.

#### Cultural and Community Approaches

If HB is related to unfair social oppression, then society has the responsibility to do something for HB patients. Society can be restructured into a better form in which traumatized or disadvantaged people are supported, suppressed people are freed and discriminated people are equally accepted. It was reported that patients with HB were helped by providing a job and social participation to improve their low self-esteem.^34^,^35^ The laws and social system should be reformed in order to help those in an inferior situation. The unfair treatment for women should be improved. Support groups, including religious organizations or other community organizations should be activated to provide a positive and compassionate environment. Christian churches in Korea are supposed to help patients with pastoral counseling through transformation of the personality structure.^58^

## Diagnosis

The diagnostic issues regarding HB are as follows: whether or not HB is a culture-related syndrome of Korea, how HB is diagnosed in the DSM-IV or International Statistical Classification of Diseases and Health-related Problems (ICD)-10 system, whether HB is a disorder or not, and lastly whether or not a new anger disorder can be formulated from the concept of HB, an anger syndrome.

### Korean culture-relatedness of hwa-byung

Though there has been debate on the issue of HB being a Korean culture-related syndrome,^69^,^70^ HB has been generally considered as a Korean culture-bound or culture-related syndrome.^1^,^5^,^10^,^20^,^49^ First of all, its name was made by ordinary people and it has been used for a long time.^69^ The term HB is found in the literature dated to the 1603 (Chosun Wang-cho Shillok, the official daily record of the Chosun dynasty).^22^ But it is not found in the classical textbooks of Korean traditional oriental medicine or those of China or Japan.^22^ The etiology of HB, in regard to anger, has also been related to the traditional and contemporary culture-related concepts of Koreans. Especially the concepts of anger-fire, ukwool/boon and haan in relation to HB have developed in the historical and cultural context of Korea. Further, the symptoms are characterized by culture-related symbolization of anger-fire and its suppression and release as well.^4^,^5^

HB is especially related to "haan" as an etiology and to the symptoms as well, by which Korean culture has been characterized,^49^-^51^ and it is assumed that HB is a pathologic form or a disease form of haan.^10^ Together with haan, there is another Korean culture-related affect called "jeong". HB has been explained in connection with jeong, haan and the we-collectivism of Koreans.^15^,^20^

Supporting the culture-relatedness of HB, Chon^71^ reported that the experience and expression of anger was different between Koreans and Americans. Also Roberts et al.^46^ assessed the features of HB with using the Minnesota Multiphasic Personality Inventory (MMPI)-2 scale and they revealed four-components that included general health, gastrointestinal symptoms, hopelessness and anger, which applied well to Koreans, but not to Americans.

Accordingly, HB seems to roughly meet the criteria of a single culture-bound syndrome, as proposed by Hughes et al.,^72^ which include ① the peculiarities of the diagnostic process used, ② the deviant aspects of the etiology of such disorders, ③ the unique meanings of the term when it is used with the term culture (i.e., culturebound) and ④ the special characteristics of the displayed symptoms.

### Diagnostic and Statistical Manual of Mental Disorders diagnosis

When patients with self-labeled HB were diagnosed according to the criteria of the DSM-III-R, many of them were diagnosed as having depressive disorders (major depression or a dysthymic disorder), an atypical somatization disorder (atypical because of including the Korean culture-related somatic symptoms of HB) and a generalized anxiety disorder (GAD) or panic disorder, with some of them classified as nitric oxide synthetase (NOS), the atypical or undifferentiated types.^2^,^3^,^9^,^13^

In the recent studies using the criteria of the DSM-IV and the diagnostic criteria of HB,^23^ Kwon et al.^18^ reported that among 118 patients with HB, major depressive disorder was diagnosed in 42 patients, dysthymic disorder was diagnosed in 9 patients, GAD was diagnosed in 22 patients, panic disorder was diagnosed in 6 patients and an undifferentiated somatization disorder was diagnosed in 10 patients, but 18 patients (15.2%) had only HB. In a similar study, Son^19^ reported that among 221 patients, HB was diagnosed with using the diagnostic criteria of HB^23^ in 74 patients and they had comorbid major depressive disorder (47.3%), dysthymic disorder (19.2%), GAD (29.1%), and somatization disorder NOS (17.4%), but 11 (15%) patients had only HB. These results are almost same with those results of the previous studies that focused on the diagnosis of patients with self-labeled HB.^2^,^3^,^9^,^13^

The fact that about 15% of HB patients are diagnosed with only HB without any other disorders suggest that the current DSM-IV seem to fail for diagnosing HB or anger syndrome because as they do not include any criteria for the evaluation of anger and the anger-related symptoms. In relation to this issue, DSM and ICD are criticized as being cultural too as they are based on a Eurocentric cultural heritage, as they are the products of a particular Euro-American biomedically-based psychiatry, and as they are dominated by the language categories and epistemologies of scientific objectivism.^73^

### Is hwa-byung a disorder?

Though HB means literally anger (fire) disease, there has been debate among Korean psychiatrists regarding if it is a true disorder. Some Korean psychiatrists have argued that HB is no more than merely a general term that means stress or a "neurotic" state.^6^ Actually, lay people use the term HB extensively to mean not only suppressed and accumulated anger, but also the bodily discomfort due to chronic stress, or a disordered state following psychological trauma. Western psychiatrists have also regarded HB as a variant of depression^42^ or a kind of somatization disorder,^74^ and they have suggested that HB could not be a disorder.^75^

However, when reviewing the studies on HB, I suggest that HB is a disorder related to anger. In the DSM-IV, "each mental disorder is conceptualized as a clinically significant behavioral or psychological syndrome or pattern that occurs in the individual and that is associated with present distress (e.g., a painful syndrome), or disability (i.e., impairment in one or more areas of functioning) or with a significant increased risk of suffering death, pain, disability or an important loss of freedom".^45^ According to this definition, HB seems to be proved to be such a syndrome or pattern, that occurs in the individual and that is associated with present distress and some extent of impairment in one or more areas of functioning. Then the concept of HB can be shaped into a disorder with using its clinically significant behavioral or psychological symptoms.

#### Construction of the Diagnostic Criteria of Hwa-Byung

In contemporary descriptive psychiatry, the symptom profile is a key component among the various clinical correlates for making the diagnosis of a disorder. Therefore, among the various symptoms that HB patients are presenting, the anger and anger-related emotional, cognitive, behavioral and somatic symptoms should be essential symptoms for the diagnosis of HB. Those symptoms should be significantly correlated with the anger state, but not with other emotions, especially depression because not only the anger attacks, but the HB too have been most frequently related with depression.^42^,^65^,^66^,^68^ Kwon et al.^28^ reported that the characteristic symptoms of HB were different from the symptoms of depressive disorders. Son^19^ had also identified the characteristic symptoms of HB, which should be included in diagnostic criteria of HB. Such symptoms included subjective anger, external anger, a heat sensation, pushing-up in the chest, dry mouth, sighing, respiratory stuffiness, hate, haan, going-out, an epigastric mass, palpitation, headache/pain, being easily frightened, many thoughts and much pleading, which have all been suggested as the typical symptoms of HB in previous studies.^4^,^14^,^18^ The symptoms that patients with HB display, but significantly less frequently than the patients with depressive disorders, included depressed mood, decreased interest, anorexia with weight changes, insomnia, retardation, fatigue, worthlessness, feelings of guilt, impaired concentration and suicidal ideas,^18^ which seem to differentiate HB from depressive disorders.

Accordingly, based on ① the clinical studies on the etiology, symptoms and prognosis of self-labeled HB,^1^-^19^ and ② the symptoms of HB^28^ which was diagnosed with the diagnostic criteria of HB^23^ and which were significantly different from those of patients with depressive disorder as diagnosed by the DSM-IV, the research criteria of HB can be constructed (Table 1).

These diagnostic criteria are now being tested for clinical practice.

These criteria are almost the same as the diagnostic criteria of HB as developed by Kim et al.^23^ But Kim's criteria is different by ① including insomnia, a mass in the mind (may be same as haan), nihilistic ideas with self-pity (may be same as haan), but Kim's criteria do not include symptoms of sighing, haan, hate, going-out, many thoughts and much pleading, ② not including the criteria that patients have to suppress their anger so as not to jeopardize harmonious social relationships in the Korean cultural context, and ③ not including the condition that the disturbance is not due to a general medical condition or the direct physiological effects of a substance, and the disturbance is not better explained by other mental disorders, and the disturbance is not merely an exacerbation of a preexisting Axis I or Axis II disorder.

## Proposing an Anger Disorder

### Hwa-byung as anger disorder

I have argued that a new concept of anger disorder can be formulated from HB, and this needs to be included in the new international classification system.^15^ The rationales for such a proposition are as follows. ① Anger-related syndrome is possibly universal. ② The international classification systems may not be adequate as they can not diagnose an culture-related anger syndrome like HB. ③ The clinical profile of HB as an anger disorder seems to be definite and it is different from depression.^28^ ④ Patients with only HB but who do not have a DSM-IV diagnosis have been identified. Patients with only HB without any DSM-IV diagnosis were diagnosed in about 15% of the patients with neurotic stress-related and somatoform disorder.^18^,^19^ If the over-inclusive concepts of the current DSM-IV criteria for depressive, anxiety and somatoform disorders are pruned to narrower concepts, then the percentage of the HB only group will increase while that of the patients in the undifferentiated, atypical or NOS categories will decrease and more patients with HB will be found to be comorbid with depressive, anxiety or somatization disorders. This issue should be explored in the future.

### Does hwa-byung or anger syndrome only exist in Korea?

After having conducted research on HB, author questioned why there is no anger disorder in the international classification of mental disorders. Is an anger syndrome like HB found only in Korea? I think perhaps not. As anger is a universal emotion and one of the basic human emotions, it is quite possible that problematic, pathological or dysfunctional anger, HB, anger disorder, or even haan can be found with different names in other cultures, wherever people are suppressed.

Nevertheless, anger and anger disorder rarely appear in the psychiatric literature. Anger has been described as a "forgotten emotion" in the psychiatric research.^76^ Why has anger disorder not been conceptualized in psychiatry? Probably there are many reasons. Anger, if not so serious, might be considered as a normal emotional reaction. Anger is generally not expressed directly because of the social stigma. The expression of anger is less acceptable in society than the expression of depression or anxiety. Expressing anger is considered as shameful in Korea too, and it is evidence for a lack of control or having personality problems (But HB is far less stigmatized in Korea.). Usually anger is soon suppressed or disguised into other clinical forms like somatic symptoms, HB or neurotic disorders. Further, patients with anger problems may not visit psychiatrists, but instead they talk to family members, friends, counselors and/or pastors. Or they may visit internists, family doctors or alternative medical practitioners only for their anger-related somatic symptoms. Further, psychiatrists themselves might have lost opportunities to identify anger problems because they might have uncons-ciously tried to avoid involvement in anger-related problems.

### Anger-related disorders in other cultures

Similar syndromes or symptom complexes related to anger have been reported in the English journals such as aggressive disorder by Yudofsky,^63^ irritability syndrome by Snaith and Taylor^76^ and Kennedy,^77^ anger attacks by Fava et al.,^64^,^65^,^68^ and aggression in personality disorders by Coccaro et al.^66^,^67^ Some of the somatic symptoms reported in anger attacks, including hot flashes, feeling hot, face getting red, chest tightness and palpitation,^64^,^65^,^68^ are quite common in HB patients. A similar disorder in the DSM-IV is intermittent explosive disorder,^44^ which is characterized by behavioral symptoms of serious physical assault and destruction of property rather than the emotion of anger. But there may be patients with intermittent anger and they do not have explosive disorder. As a culture-related syndrome, ataques de nervios of Mexico is known as an acute anger syndrome.^78^

Anger problems have been reported in cultures other than western countries.^79^-^81^ Neurasthenia is known to include as its culture-related symptoms "huoki da" meaning an excess of energy and "cold fire" in the body.^82^ Like HB, anger problems are found more often in women in relation to marriage in other cultures.^79^,^83^ Some of the symptoms of "trapped housewife syndrome" of the USA seem to be very similar to those of HB of Korean housewives.

In contrast to psychiatrists, many clinical psychologists in USA have conceptualized clinical models of problematic anger and they have suggested cognitivebehavioral treatment for such a condition. Spielberger^84^ defined anger as AHA! Syndrome, including anger, hostility and aggression, and he classified anger into state anger, trait anger, anger-in, anger-out and anger-control. Deffenbaucher^85^,^86^ has argued that dysfunctional anger has been hided in various DSM diagnoses, including depressive disorders, schizophrenia, mania, delusional disorders, impulse control disorders, antisocial personality disorder, organic mental disorders (epilepsy) and posttraumatic stress disorder (PTSD), and that anger problems should be treated inependently from other emotional problems.

### Concepts of a new anger disorder

Clinical experiences with patients with anger or aggression have suggested to conceptualize anger disorder or aggressive disorders. Yudofsky et al.^63^ argued that, just as anxiety and depression may be conceptualized either as symptoms or as specific disorders, aggressive behavior may be distinguished as a symptom or as a distinct disorder and proposed the specific diagnostic category of organic aggressive disorder. In this regard, I am suggesting to focus on anger emotion, which is behind or accompanied by aggressive behavior, rather than aggression.

Allover the world, a certain group of patients may visit psychiatrists with anger-related problems and symptoms such as HB, anger attacks, irritability or aggressive beavior without depressive disorders, or they have a mild form of intermittent anger-explosive disorder without seious acts of assault or destruction of property. Such conitions may then deserve to be renamed as anger disorder in the diagnostic system. Theoretically, anger disorder is coneptualized for an angry mood as depressive disorders are conceptualized for a depressive/sad mood, anxiety disorers are conceptualized for an anxious mood, fear is coneptualized for a phobic mood, and mania and hypomania are conceptualized for a pleasurable mood.

To make a culture-bound syndrome into a diagnostic term, it has been suggested to propose the diagnostic criteria for that condition in universal language that can be applied to various cultural communities.^72^ In the case of HB, if the culture-related components of HB are removed, translated or modified into general terms, then the concept of anger disorder will be formulated. This construction of the concept of anger disorder is beyond the cultural-relatedness of HB^15^ and this can serve as a good example of the conceptualization of a new disorder from several culture-bound syndromes. Here is a case of HB that was diagnosed as anger disorder.

#### A Case History of Anger Disorder

The patient is 31 year old man. His first complaints during the psychiatric interview were pent-up anger and irritability. He said his problems might be trivial, but it was very hard for him to tolerate the anger that was boiling-up from inside. Yet he has not shown any violent behavior. He also complained of sleepiness and being tired, but not of any pain on the body. But his mind was so painful because of intolerable anger that persisted from the morning to night. He wanted to be happy as his first son was born last year. But he could not feel happy at all because of anger. As he had no job, he was uncertain about his future. His father had recently been taking care of him and his family. Now the problem was that the relationship between him and his father wasn't good. Since childhood, he hated his father, who had been so rigid and suppressing in personality. His father used to drink much and beat his wife and children and destroy property. The patient, when younger, had to restore all these destroyed things. His clever younger brother had left home earlier, but the younger sister was so weak and had to be protected by the patient. Nevertheless, his mother had shown support for the father because she had been disciplined to behave that way since her childhood according to Korean cultural Confucian tradition and because she was so weak as well. He was deeply disappointed whenever he had to kneel before his father for forgiveness following his mother's pleading after the patient verbally argued with his father. Whenever his mother was hospitalized after being beaten by father, he had felt impulses to kill his father. His bad relationship with his father has worsened when he discovered his father's extramarital affair and he let his mother know about it. Since then he had endured his parents' frequent quarrels. He asked many times for mother to divorce his father, but she refused, saying "anyway he is your father" or "nothing will be left for living and taking care of children after divorce". Now his anger is especially provoked by seeing father touching his baby. Whenever his father touched his baby without washing hand or after smoking, he felt murderous hostility and anger. The anger and hatred were so strong that he felt almost crazy at that moment. But he could not show any protesting behavior or express anger. He used to go-out to avoid distress. While he talked about this, his voce was calm, but he was tremulous and showed tears in eyes and sighing. Now, what is more distressing is that he can not hate his father any more because his father was found to have cancer of the prostate. The doctors said his father had 3 years to live. He is now responsible to show traditional piety to his father as a son. He said all his efforts must be in vain.

This patient is better diagnosed as having anger disorder rather than HB because he has suffered from only pent-up anger and psychological distress but not culture-related classical symptoms of HB including epigastric mass, pushing-up, hot sensation, respiratory stuffiness, ukwool/boon or haan.

### The proposed diagnostic criteria of anger disorder

Anger is experienced everyday in a number of interpersonal, family and occupational situations and, when it is short-lived and of low intensity, it may be normal, positive and of some help with stimulating vitality as stress or haan may do. Yet when anger is persistent and intense, it may be disruptive and it may then be considered as disordered. The question is: when is anger an abnormal or disordered emotion? Anger may be disordered according to whether anger is "associated with present distress or disability or with a significant increased risk of suffering death, pain, disability or an important loss of freedom."^45^ This disordered, pathological or dysfunctional anger includes the anger in HB and anger attacks.

For anger disorder to be an independent diagnostic category, the research diagnostic criteria should first be established for anger disorder in a universal language. Second, an international cross-cultural study for evidence should be conducted with the same protocol.

What symptoms should be included in the soon-to-be proposed diagnostic criteria of anger disorder? Anger should first be defined. In this regard, the symptoms of HB,^4^,^10^,^18^,^19^ AHA! Syndrome,^84^ anger attacks^64^,^65^,^68^ and dysfunctional anger^85^ may be considered as good candidates. Spielberger^84^ developed the concept of the AHA! syndrome to illustrate the assumed interconnections among the emotion of anger, a hostile attitude and aggressive behavior. The basic assumption behind AHA! syndrome is that anger is always at the core of expressed hostility and aggression. The symptoms of anger attacks include sudden spells of anger and more than 4 symptoms of 13 symptoms, including palpitation, flushing, chest tightness or pressure, paresthesia, lightheadedness or dizziness, excessive sweating, shortness of breath, shaking or trembling, intense fear or anxiety, feeling out of control, feeling like attacking others, physically and verbally attacking others and throwing or destroying objects.^68^ Dysfunctional anger, as described by Deffenbacher,^86^ is a syndrome comprised of: ① emotional experience (e.g. feeling angry or furious), ② psychophysiological arousal (e.g. short, rapid breathing, a hot sensation and increased muscle tone, such as a clenched jaw), ③ cognitive processes (e.g. hostile attributions, images of revenge and retaliation, labeling as fire)(similar to many thoughts in HB), and ④ individual distress leading to significant adverse consequences (e.g. damaged relationships, impaired work performance and legal problems). The symptoms profile should also be different from that of depression because anger has been closely related to depression.^59^,^60^,^63^,^65^,^68^

#### The Research Diagnostic Criteria of Anger Disorder

Based on a review of the studies on various anger syndromes, the formulation of a new concept of anger disorder is proposed as follows. ① The emotional symptom of anger: arousal of anger as a symptom. ② Psychological cognitive symptoms: perception of unfairness, hate/hostility and many thoughts (obsessiveness, harboring hate or ideas of revenge). ③ The physiological phenomena of arousal accompanied with anger: palpitation, a heat sensation, dry mouth and muscular tension. ④ Anger-specific behavioral symptoms: irritability, and aggressive behavior (aggressive facial expression, violence, abuse, fighting or destruction of property). ⑤ Anger-specific somatization symptoms: respiratory difficulties of stuffiness or rapid, short breathing, and headache/pain. ⑥ The anger disorder is with or without a comorbid sad and depressive mood with tears or an anxiety state with agitation. This anger disorder may include a chronic form like HB, an acute form and intermittent forms like anger attacks and intermittent explosive disorder with or without the expression of violent behavior.

Research on anger disorder should be done with using these research criteria for its prevalence, etiology, symptoms and their evaluation (via an anger scale), the cormobidity, the differential diagnosis, treatment, clinical course and prognosis.

### Where is anger disorder in the international classification of mental disorders?

#### Anger Disorder in the Current DSM-IV or ICD-10

In the ICD-10, anger disorder may be classified as F46 Anger disorder in the category of F40-F48 neurotic stress-related and somatoform disorders, or as F48.2 Anger disorder in the category of F48 other neurotic disorder. In the DSM-IV, anger disorder is classified as 300.XX Anger disorder.

#### Anger Disorder in the New DSM-V and ICD-11

If anger disorder is not to be included as an independent category in the new classification system, a larger category of mood disorder can be considered. Then, anger disorder may be included as one disorder in this larger category of mood disorders, which include depressive and anxiety disorders as well. This suggestion is based on integrating ① the relationship of anger (including HB) with depression and anxiety,^59^,^60^ ② the concept of mixed anxiety-depression,^87^ ③ even intermittent explosive disorder being frequently comorbid with mood and anxiety disorders.^88^ Even mania/hypomania may be added in this larger category as they frequently accompany anger and aggressive behavior.

This idea seems to be reasonable because of the theoretical dynamic inter-relationship among anger, depression, anxiety and excitement. Further, there has been empirically noted the high comorbidity among these anger, depressive and anxiety states, while they are differentiated each other by factor analysis with rated symptoms.^89^ Also, van Praag has recently suggested a type of depression called as anxiety/aggression (anger)-driven depressions of 5-HT-related depression, in which dysregulation of anxiety and/or aggression (anger) are primordial and mood-lowering is a derivative phenomenon. Thus, they may all be considered as part of a larger group of mood disorders. The theoretical frame of this larger category seems, if manic disorder is excluded, to be similar to the concept of the neurotic, stress-related and somatization disorders of F40 in the ICD-10 or the adjustment disorder with a predominant mood in the DSM-IV. Likewise in this larger mood disorder, subcategories may be named according to the predominant mood. Mood disorder with a predominant depressive mood includes major depressive disorder, dysthymic disorder and atypical depression, and mood disorder with predominant anxiety includes GAD, Phobia, PTSD and panic disorder. Mood disorder with predominant anger includes acute anger disorder, chronic hwa-byung and intermittent anger-explosive disorder. Or mood disorder with predominant pleasurable affect may be included in this category including bipolar disorder, mania and hypomania. These mood disorders may have psychotic features, including delusion or hallucination. This classification may help to overcome the complicating issues related to the high comorbidity among mood disorders including depressive, anxiety and manic disorders.

### The advantages of having anger disorder

Anger is known to be one of the most fundamental human emotions, and it is considered to be a vital factor of health, stress and disease. Recent research has suggested that anger is one of the most critical psychological problems involved in physical disorders, including cardiovascular diseases, cancer, diabetes and pain.^91^-^93^ HB has been known to Korean as a chronic illness that may lead patients to death. Further, anger is being revealed as an important emotional reaction related to nursing^29^ and giving care to patients with chronic illness.^44^

If anger disorder is included in the official classification system and this is known to the public, then it may facilitate the early detection and early treatment of anger problems and it can prevent further development of more serious psychiatric or physical disorders related to anger. It will promote scientific research on the anger related to desperate illness or dying, the anger of people who give care to chronically ill patients, the anger related to social violence and the anger of its victims, and hopefully inter-ethnic rage and terrorism. It will also stimulate scientific research on the biological nature of anger and the pharmacological treatment of anger as well.

### International collaborating studies

#### For Evidence

International comparative studies are needed to gather evidence of this anger disorder, including not only clinical correlations of the disorder, but also its bio-psycho-social etiological basis and treatment. The areas of international comparative studies include not only the clinical correlations of the disorder, but also the biological, psychological and social aspects of anger disorder and treatment. For these collaborative studies, information should be collected from all over the world regarding anger syndrome with using common research tools, including the agreed-upon diagnostic criteria of anger disorder. I have proposed the research diagnostic criteria of anger disorder in this paper for conducting these collaborating studies.

#### Treatment of Anger Disorder

The psychosocial treatment for anger disorder may be similar to that for HB and this was suggested in the section on the treatment of HB. In psychotherapy, the patients may be asked to explore the root causes of their anger, or they may be comforted with an emphasis on understanding, forgiving and reconciliation, or resignation. Marital and family therapy is recommended for solving familial conflicts. Patients may receive education for anger management or they may undergo cognitive behavioral treatment.^85^ As in HB, the methods of healing including music, dance and festive activities, or meditation may be applied for the treatment of anger.

For drug treatment, selective serotonin reuptake inhibitors (SSRIs) have been reported to be effective to control anger, aggressive outbursts^64^-^66^ and even hostility.^94^ In a previous study, SSRIs and fluoxetine significantly improved post-stroke emotional incontinence and anger, but not post-stroke depression.^95^ There have been many studies on the relationship between aggression (including violence, impulsivity, suicide and anger) and serotonergic abnormalities in the brain.^96^,^97^ Accordingly, one of the future directions for the study of anger disorder might be to investigate the relationship between anger disorderand the central serotonin system and the treatment of anger syndrome with using serotonin-related drugs such as serotonin reuptake inhibitors.^64^-^66^,^95^,99 New drugs may be hopefully be developed by this research.

Anticonvulsants have been recommended for aggressive behavior.^63^,^67^ The physical symptoms of HB and anger disorder such as palpitation, hot flushing or a heat sensation may be relieved with beta-blockers or antiadrenergic agents. Hypnotics can be used for treating insomnia. Comorbid anxiety and agitation may be helped by anti-anxiety drugs. Bodily pains, indigestion and other physical symptoms may be well controlled with conservative medication.

## Conclusion

The rationale for proposing this new anger disorder is first that patients with only anger syndrome like HB are visiting psychiatrists for help, and second that the symptoms and the other clinical correlates of this anger syndrome are different from at least the depressive disorders. In the new classification system, anger disorders should be included as an independent category or they may be included as one of the mood disorders, which include depressive disorders, anxiety/phobia or even manic disorder. International collaborative study is needed to identify such anger disorders in various cultures, to refine the concept of anger disorder, to find treatment methods for anger problems and to explore the biological basis of this disorder.

## Figures and Tables

**TABLE 1:**
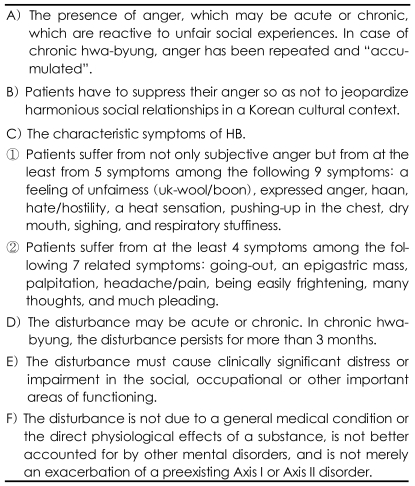
The research diagnostic criteria of hwa-byung
